# Genomic insights into *Mycobacterium simiae* human colonization

**DOI:** 10.1186/s40793-017-0291-x

**Published:** 2018-01-08

**Authors:** José L. Steffani-Vallejo, Marion E. Brunck, Erika Y. Acosta-Cruz, Rafael Montiel, Francisco Barona-Gómez

**Affiliations:** 1 Evolution of Metabolic Diversity Laboratory, Unidad de Genómica Avanzada (Langebio), Cinvestav-IPN, Irapuato, Mexico; 2 Paleogenomics Laboratory, Unidad de Genómica Avanzada (Langebio), Cinvestav-IPN, Irapuato, Mexico; 3 Centro de Biotecnología FEMSA, Escuela de Ingeniería y Ciencias, Tecnológico de Monterrey, Monterrey, Mexico; 4 Present address: Laboratorio de Biología Molecular, Facultad de Ciencias Químicas, Universidad Autónoma de Coahuila, Saltillo, Mexico

**Keywords:** *Mycobacterium simiae*, Nontuberculous mycobacteria, Opportunistic pathogen

## Abstract

*Mycobacterium simiae* (Karassova V, Weissfeiler J, Kraszanay E, Acta Microbiol Acad Sci Hung 12:275-82, 1965) is a slow-growing nontuberculous *Mycobacterium* species found in environmental niches, and recently evidenced as an opportunistic Human pathogen. We report here the genome of a clinical isolate of *M. simiae* (MsiGto) obtained from a patient in Guanajuato, Mexico. With a size of 6,684,413 bp, the genomic sequence of strain MsiGto is the largest of the three *M. simiae* genomes reported to date. Gene prediction revealed 6409 CDSs in total, including 6354 protein-coding genes and 52 RNA genes. Comparative genomic analysis identified shared features between strain MsiGto and the other two reported *M. simiae* genomes, as well as unique genes. Our data reveals that *M. simiae* MsiGto harbors virulence-related genes, such as *arcD*, ESAT-6, and those belonging to the antigen 85 complex and *mce* clusters, which may explain its successful transition to the human host. We expect the genome information of strain MsiGto will provide a better understanding of infective mechanisms and virulence of this emergent pathogen.

## Introduction

Contrasting with the declining incidence of *Mycobacterium tuberculosis*-caused tuberculosis, the increasing number of nontuberculous mycobacteria infections is concerning. Amongst NTM, the *Mycobacterium simiae* complex contains 19 species [1], including *M. simiae* [2], which is considered the most important species in terms of its clinical relevance [3]. *M. simiae* is a slow-growing saprophyte that has been isolated from several environments including water and soil [3, 4]. In addition to thriving in environmental niches, *M. simiae* has been associated to infections in both immunocompromised [4, 5] and immunocompetent patients [6], with clinical cases reported worldwide [5, 6]. Thus, *M. simiae* is considered an emergent pathogen [7–9]. Previous sequencing efforts have provided draft genome sequences for *M. simiae*, namely, strains MO323 (accession PRJNA276839) and DSM 44165 (accession PRJEB1560) [10], which were isolated in early years from the United States of America (1989) and India (1965), respectively. We expand here on this genomic data by reporting the genome sequence of a clinical isolate from a Mexican patient (bronchial lavage) obtained in 2011, designated as MsiGto, which is approximately 745 Kbp and 901 Kbp larger than the previously published *M. simiae* genomes, respectively. Additionally, we perform a comparative analysis between the genomes of strain MsiGto and the previously reported DSM 44165 and MO323 strains to provide insights that could unearth the transition from environmental bacteria to pathogenic NTM organisms.

## Organism information

### Classification and features

**Fig. 1:**
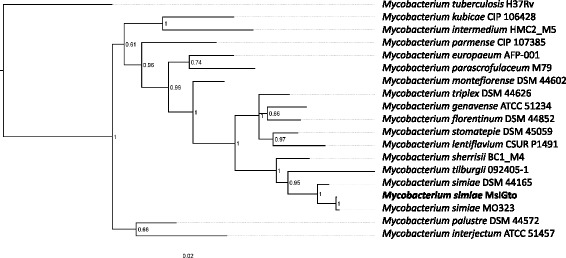
Phylogenetic tree showing the relationship of *M. simiae* MsiGto with selected species members of the *M. simiae* Complex, including 2 relevant *M. simiae* strains. Phylogenetic reconstruction was obtained using Bayesian inference. Numbers at the nodes are the values of posterior probabilities. The tree was obtained after 1 million generations with mixed model. Sequence data from *Mycobacterium tuberculosis* H37Rv was used as an outgroup

To identify strain MsiGto as an *M. simiae* isolate, typical DNA markers for mycobacterial identification, such as the 16S rRNA, *rpoB* and *hsp65* genes [11–13] were localized on the sequenced MsiGto genome, and compared to other publicly available sequences using BLASTN on its default settings. The MsiGto 16S rRNA gene exhibited 100% identity with previously deposited *M. simiae* sequences (strains DS39, DS34, DS31, DS24, DS19, DS3, DS2, MO323 and ATCC 25275). The MsiGto *rpoB* gene sequence showed 99% identity with *M. simiae* MO323. Similarly, there was 100% identity between the MsiGto and MO323 *hsp65* genes. For comparison, a BLAST of these markers from DSM 44165 and MO323 evidenced a similar trend with 99% identity for the 16S rRNA gene and 100% identity for *rpoB* and *hsp65*. Figure 1 shows the phylogenetic position of *M. simiae* MsiGto within the *M. simiae* complex, based on a concatenated gene tree including the sequences of the 16S rRNA, *rpoB* and *hsp65* genes (1507 bp in total).

Further analysis such as the Average Nucleotide Identity and Average Amino Acid Identity, preformed as previously reported [14], confirmed the high resemblance between *M. simiae* MsiGto and *M simiae* strains MO323 and DSN 44165 (Table 1). The ANI values were above 95% in all cases, indicating that the compared organisms belong to the same species. Similar results were found using Genome-to-Genome Distance Calculator [15, 16] with a calculated distance of 0.0292 and a DNA-DNA hybridization estimate of 77.40% using the formula for draft genomes. These results provide a taxonomic background in which the genome insights presented in the following sections can better accounted for, namely, large genotype differences of MsiGto, despite close taxonomic relationships with DSM 44165 and MO323 strains.

**Table 1:** Average Nucleotide Identity (A) and Average Amino acid Identity (B) between *M. simiae* MsiGto and other *M simiae* strains sequenced to date

Organism	M. simiae MsiGto A/B	M. simiae DSM 44165	M. simiae MO323
M. simiae MsiGto	–	97.25/ 97.51	98.99/98.95
M. simiae DSM 44165	97.25/ 97.51	–	97.26/97.59
M. simiae MO323	98.99/98.95	97.26/97.59	–

## Genome sequencing information

### Genome project history

Despite *M. simiae* being one of the most relevant NTM due to its emergence as a human pathogen, the genomic features and genetic potential of this species remain poorly described. In collaboration with the State Laboratory of Public Health of Guanajuat, located in central Mexico, we had access to a clinical isolate of *M. simiae*, termed MsiGto. The sample was isolated from a 90 years old female patient from León, the largest and most industrialized city of the State of Guanajuato, México, in 2011 (Table 2). This sample was selected for genomic sequencing due to the emerging clinical importance of strains from the *M. simiae* complex worldwide [8, 9, 17–19], combined with a lack of representative genomic sequences from Mexico to this date. The available genome sequences could allow comparative analyses in order to increase our understanding of this opportunistic bacterium.

**Table 2:** Classification and general features of *Mycobacterium simiae* MsiGto [51]

MIGS ID	Property	Term	Evidence code^a^
	Classification	Domain *Bacteria*	TAS [52]
		Phylum *Actinobacteria*	TAS [53]
		Class *Actinobacteria*	TAS [54]
		Order *Actinomycetales*	TAS [55, 56]
		Family *Mycobacteriaceae*	[54, 56, 57]
		Genus *Mycobacterium*	TAS [58, 59]
		Species *Mycobacterium simiae*	IDA
	Gram stain	Weakly Postive	IDA
	Cell shape	Irregular rods	IDA
	Motility	Non Motile	IDA
	Sporulation	Nonsporulating	NAS
	Temperature range	Mesophile	NAS
	Optimum temperature	37 °C	NAS
	pH range; Optimum	5.5–8; 7	IDA
	Carbon source	Starch	IDA
MIGS-6	Habitat	Human Associated	NAS
MIGS-6.3	Salinity	Normal	NAS
MIGS-22	Oxygen requirement	Aerobic	NAS
MIGS-15	Biotic relationship	Parasitic	IDA
MIGS-14	Pathogenicity	Pathogenic	NAS
MIGS-4	Geographic location	Mexico/Guanajuato	NAS
MIGS-5	Sample collection	2014	NAS
MIGS-4.1	Latitude	Not Reported	NAS
MIGS-4.2	Longitude	Not Reported	NAS
MIGS-4.4	Altitude	Not Reported	NAS

^a^Evidence codes - *IDA* Inferred from Direct Assay, *TAS* Traceable Author Statement (i.e., a direct report exists in the literature), *NAS* Non-traceable Author Statement (i.e., not directly observed for the living, isolated sample, but based on a generally accepted property for the species, or anecdotal evidence). These evidence codes are from the Gene Ontology project [60]

### Growth conditions and genomic DNA preparation

MsiGto was isolated from a sputum specimen. Briefly, 2 mL sample were transferred to a sterile tube and decontaminated by adding an equal volume of 4% NaOH with phenol red. The mix was immediately vortexed and incubated at 37 °C for 15 min. The liquefied sample was centrifuged at 3000 g at 4 °C for 15 min, the supernatant was discarded and pH was adjusted between the 6.5 and 7.2 range using 1 N HCL. Finally, 0.2 mL of sample were inoculated into Lowenstein-Jenssen slants (Difco) and incubated at 37 °C for three weeks.

Biomass was collected and suspended in phosphate buffered saline solution, pH 7.4, and inactivated by heating at 80 °C for 45 min. After centrifugation, genomic DNA was extracted from the pellet using the FastDNA SPIN Kit for Soil (MP Biomedicals) according to manufacturer’s instructions. Extracted gDNA was assessed for quantity and quality using a NanoDrop Spectrophotometer (Thermo Fisher Scientific).

### Genome sequencing and assembly

The MsiGto genome was sequenced on a HiSeq 2000 platform with a 100 bp paired-end cycle according to standard Illumina protocols, at Macrogen facilities. Quality of the generated sequencing reads (13,676,836 reads, total read length: 1,381,360,436 bp) was checked with FastQC. Reads were filtered before assembly, such that both sequences in paired-end reads exhibited more than 90% bases of quality greater than or equal to Q20. Post-filtering Q20% was 97.69 and Q30% was 88.6 at the base level. The filtered reads were assembled using SOAP de novo aligner [20], yielding 50 contigs. Scaffolding was performed with SSPACE Standard [21] and resulted in 12 scaffolds, generating a genome size of 6.7 Mb with a coverage of 216X (Table 3).

**Table 3:** Project information

MIGS ID	Property	Term
MIGS 31	Finishing quality	Draft
MIGS-28	Libraries used	Paired End Illumina
MIGS 29	Sequencing platforms	Illumina HiSeq 2000
MIGS 31.2	Fold coverage	216
MIGS 30	Assemblers	SOAPdenovo
MIGS 32	Gene calling method	RAST
	Locus Tag	B5M45
	Genbank ID	MZZM00000000
	GenBank Date of Release	April 17, 2017
	GOLD ID	Ga0183212
	BIOPROJECT	PRJNA378996
MIGS 13	Source Material Identifier	*Mycobacterium simiae* MsiGto
	Project relevance	Medical, Evolutionary

### Genome annotation

Gene prediction and functional annotation were performed using the Rapid Annotation using Subsystem Technology platform [22]. A prophage region prediction was completed using PHAST [23]. CRISPRs were searched using the CRISPR finder, and antibiotic resistance genes were investigated using Resistance Gene Identifier from the Comprehensive Antibiotic Resistance Database [24]. Genome-to-genome comparisons were performed using multiple approaches, including conservation analysis of protein families across genomes with the Protein Family Sorter from the PATRIC Platform [25], and comparing functionally related clusters using the function-based comparison of the RAST server. MsiGto and related genomes were also aligned using a genome-wide BLAST comparison, and visualized through the Artemis Comparative Tool [26] for manual inspection.

## Genome properties

**Fig. 2:**
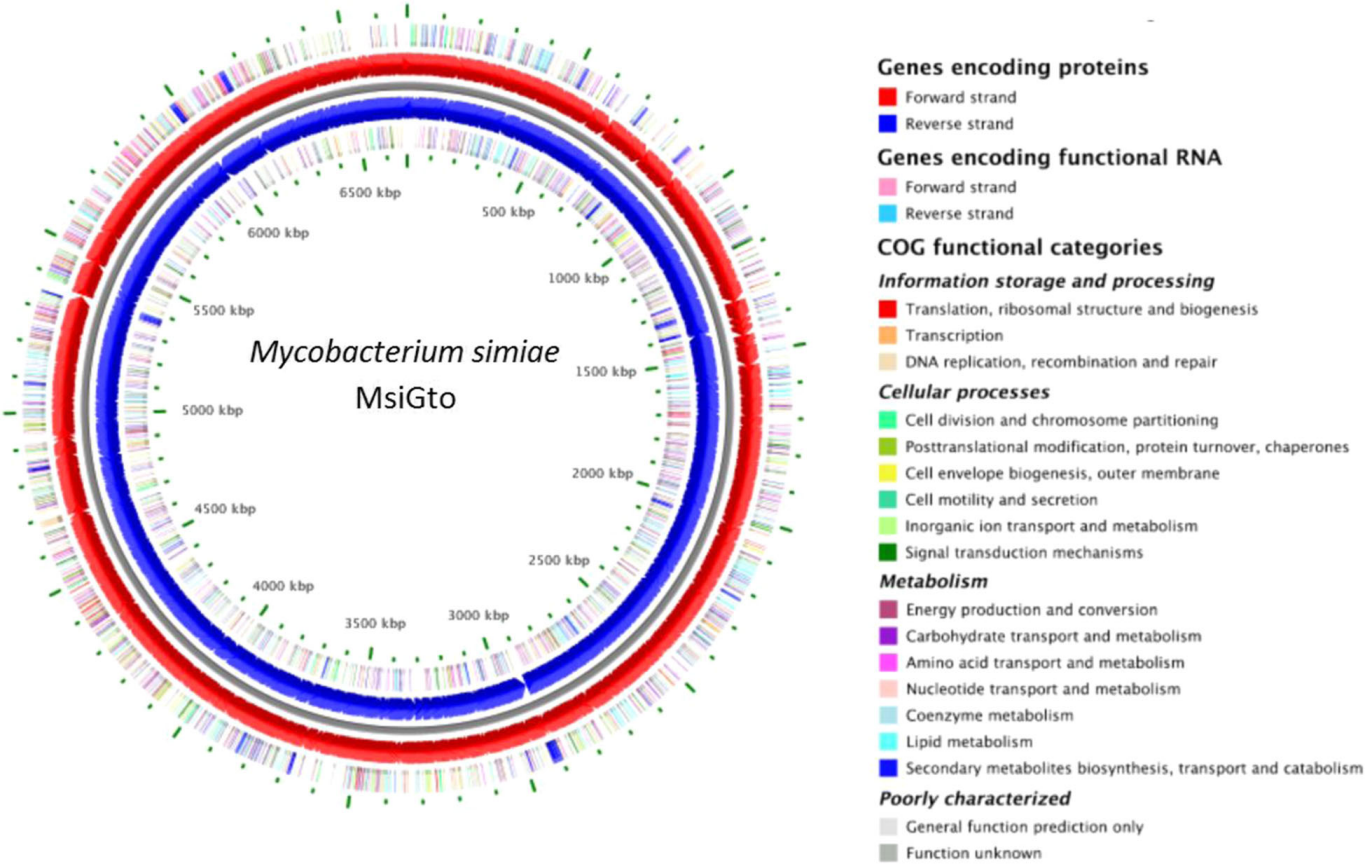
A graphical circular map of the *M. simiae* MsiGto genome keyed to the COGS functional categories. The circular map was generated using BASys web server [61]

Genomic assembly yielded a total length of 6,684,413 bp fragmented in 50 contigs, the largest *M. simiae* genome reported to date. The MsiGto genome consists of a unique chromosome, as no plasmid DNA was found. The GC content of the genome was 66.08%, consistent with other reported *M. simiae* strains. Gene prediction analysis documented 3 rRNAs, 49 tRNAs and 6409 coding sequences. Out of these, 4462 genes (69.62%) were assigned to putative functions, and 3669 genes (approximately 62.57%) were assigned to clusters of orthologous groups functional categories. Sequence searches using RGI evidenced resistance genes to amynoglycosides, ethambutol and beta lactams, which is a concern as to date standard antibiotic regimes for this species include the use of ethambutol, the aminoglycoside amikacin, as well as the macrolides azithromycin and clarithromycin [27]. The genome properties and statistics are summarized in Tables 4 and 5. Gene distribution among the COG functional categories is shown in Table 6. A circular map of MsiGto chromosome is provided in Fig. 2.

**Table 4:** Summary of genome: one chromosome, no plasmids

Label	Size (Mb)	Topology	INSDC identifier	RefSeq ID
Chromosome	6,684,413	Circular	GenBank	NZ_MZZM00000000.1

**Table 5:** Genome statistics

Attribute	Value	% of Total
Genome size (bp)	6,684,413	100
DNA coding (bp)	5,978,008	89.43
DNA G + C (bp)	4,416,391	66.07
DNA scaffolds	15	100
Total genes	6369	100
Protein coding genes	6299	99.90
RNA genes	70	1.10
Pseudo genes	160	2.51
Genes in internal clusters	579	9.09
Genes with function prediction	4713	74.00
Genes assigned to COGs	5272	82.29
Genes with Pfams domains	5009	78.19
Genes with signal peptides	260	4.08
Genes with transmembrane helices	1292	20.29
CRISPR repeats	15

**Table 6:** Number of genes associated with general COG functional categories

Code	Value	%age^a^	Description
J	161	2.53	Translation, ribosomal structure and biogenesis
A	21	0.33	RNA processing and modification
K	448	7.05	Transcription
L	192	3.02	Replication, recombination and repair
B	1	0.01	Chromatin structure and dynamics
D	55	0.86	Cell cycle control, Cell division, chromosome partitioning
V	47	0.73	Defense mechanisms
T	218	3.43	Signal transduction mechanisms
M	159	2.50	Cell wall/membrane biogenesis
N	57	0.89	Cell motility
U	36	0.56	Intracellular trafficking and secretion
O	153	2.41	Posttranslational modification, protein turnover, chaperones
C	447	7.03	Energy production and conversion
G	243	3.82	Carbohydrate transport and metabolism
E	298	4.69	Amino acid transport and metabolism
F	82	1.29	Nucleotide transport and metabolism
H	210	3.30	Coenzyme transport and metabolism
I	558	8.78	Lipid transport and metabolism
P	239	3.76	Inorganic ion transport and metabolism
Q	494	7.77	Secondary metabolites biosynthesis, transport and catabolism
R	795	12.51	General function prediction only
S	358	5.63	Function unknown
–	1082	17.03	Not in COGs

^a^The total is based on the total number of protein coding genes in the genome

## Insights from the genome sequence

### Extended insights

**Fig. 3:**
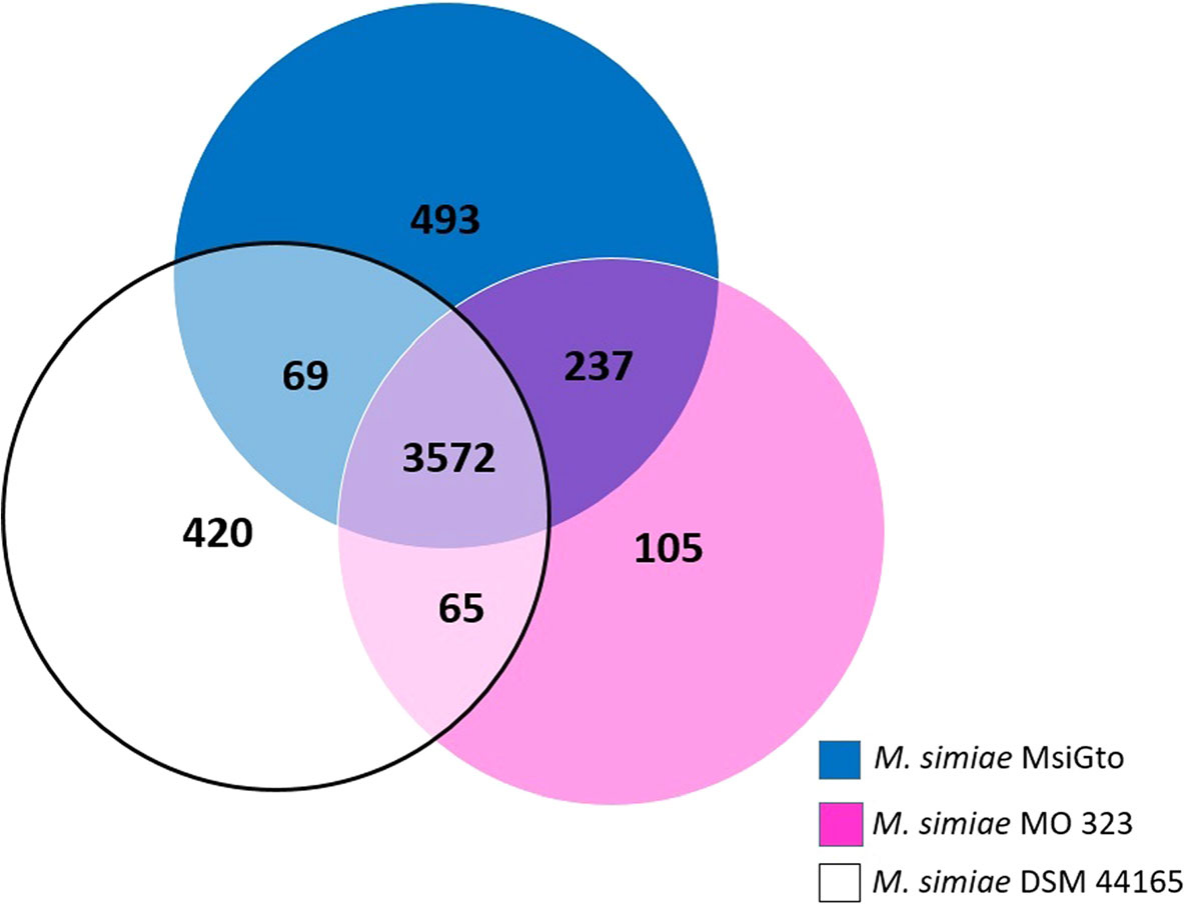
Venn diagram analysis showing the number of unique and shared family proteins as evidenced using PATRIC, between the *M. simiae* strains MO323, DSM 44165 and MsiGto

We report here the third genome sequence of *M. simiae*. MsiGto was isolated from a clinical sample obtained from an elderly woman, and is the largest *M. simiae* genome reported to date with an approximate 745 Kbp additional to the next largest genome (strain MO323). As pathogenic mycobacteria tend to undergo genome decay [28, 29], the large genomic size of MsiGto speaks out of an organism capable of thriving in both environmental conditions and in the human host. Indeed, by comparison, the genome of *M. tuberculosis*, an obligate pathogen, is significantly smaller with 4.41 Mb [30]. Meanwhile, with 6406 genes, the genome size of MsiGto is similar to that of the opportunistic pathogen *Pseudomona aeruginosa* (5570 predicted ORFs), which thrives in a variety of environments including soil, water, as well as the multiple human tissues it infects [31]. Analysis of the *P. aeruginosa* genome evidenced its large size arose from genetic expansion to enhance functional diversity rather than from gene duplication [32]. Interestingly, investigating protein families in MsiGto, in comparison with MO323 and DSM 44165, evidenced a larger proportion of proteins uniquely found in MsiGto (1.17 times more than DSM 44165 and 4.7 times more than MO323), suggestive of a relatively increased versatility in accordance with the multiple niches it can thrive in (Fig. 3).

The genome of MsiGto is rich in virulence factors, genes conferring infective mechanisms, and host immune response evasion systems. Some of these proteins are common to other pathogenic mycobacteria, and shared by the two previously sequenced *M. simiae* genomes, such as ESAT-6, known to modulate host immune responses by affecting human T-cell responses [33, 34]. Interestingly, all *M. simiae* strains including MsiGto have three of the four antigen 85 complex genes (*fbpA*, *fbpC*, and *fbpD*) responsible for cell wall synthesis. The enzyme products of these genes are responsible for the conversion of trehalose monomycolate (TMM) into the Cord Factor trehalose dimycolate (TDM) [35], which is considered one of the most important virulence factors of mycobacteria [36]. Other genera belonging to the *Mycobacteriaceae* family that include both pathogenic and environmental species, such as *Rhodococcus* [37], *Corynebacterium* and *Nocardia* [38], also produce TDM [39].

Mammalian Cell Entry proteins are cell surface exposed proteins that play a crucial role in *M. tuberculosis* virulence by permitting the bacteria to enter mammalian cells and survive inside the macrophage, modulating the immune response [40, 41]. Mce clusters consist of 4 homologous operons in *M. tuberculosis* (*mce1*, *mce2*, *mce3*, *mce4*) with a similar arrangement: two genes encoding integral membrane proteins followed by six *mce* genes (A, B, C D, E and F) [40]. Mce proteins are also involved in lipid metabolism, acting as transporters and allowing cholesterol degradation to free carbon and energy for use by *M. tuberculosis* [41]. In the genome of MsiGto we found that the cluster *mce3* is overrepresented. While the DSM 44165 and MO323 genomes present none and a single copy of mce3, respectively, two complete copies of the *mce3* cluster were found in MsiGto. As the transition from an environmental organism to a pathogen has been associated with the acquisition of Mce genes in actinobacteria [42], and Mce3 proteins are expressed by *M. tuberculosis* during the infection phase [43], it is tempting to speculate the increased number of Mce3 copies found in MsiGto provided the strain with human infection potential.

The presence of multiple copies of these potential lipid transporters in mycobacterial genomes is consistent with the finding that pathogenic mycobacteria switch from carbohydrates to lipids as their main carbon and energy source inside cells [44]. The evolution of this locus, through duplication and divergence, has almost certainly contributed to virulence in mycobacteria, and even in other distantly related actinobacteria, such as *Streptomyces* [42, 45]. Given that the ability to acquire cholesterol from the host is crucial to maintain a chronic infection, we postulate that cells having a large *mce* copy number, such as that found in MsiGto, may potentially evolve pathogenicity relatively faster when compared with other environmental mycobacteria.

A total of 493 protein families were found exclusively present in MsiGto when compared to the other two available *M. simiae* genomes (MO323 and DSM 44165). Consistent with findings in the *Pseudomonas aeruginosa* [46] genome [32], it seems that MsiGto has undergone genome expansion. Within the gene pool unique to MsiGto we found the arginine/ornithine antiporter gene *arcD*, which is involved in the persistence of the zoonotic pathogen *Streptococcus suis* [47] in host cells [48]. Additional unique genes found in the MsiGto genome participate in aromatic amino acid metabolic pathways. For instance, 2-oxo-hepta-3-ene-1,7-dioic acid hydratase (*hpcG* gene, EC 4.2.1.80) participates in the degradation of tyrosine and the 2-keto-3-deoxy-D-arabino-heptulosonate-7-phosphate synthase I alpha participates in the synthesis of chorismate (*aroG* gene, EC 2.5.1.54). The latter is an interesting observation as tyrosine is a key nutrient source during infectious growth within macrophages of some pathogenic fungus [49, 50]. At this stage, however, it would be risky to rule out the involvement of these specific genes in important environmental functions that allow MsiGto to survive outside the host, or during both lifestyles.

## Conclusions

*Mycobacterium simiae* is an organism of interest for genomic studies due to the scarce genomic data available, and its recent emergence as a human pathogen. Here we present the largest genome sequence of this species to date. The genome of *M. simiae* MsiGto presents characteristics in accordance with its adaptation to infect the Human host, with the presence of numerous virulence genes, plus some specific features that deserve further investigation. Additional *M. simiae* genomes, from both environmental and clinical isolates, should be sequenced to provide a wider evolutionary picture with functional implications. Indeed, our comparative analysis helps to better understand the evolution of host-pathogen interactions, and the molecular mechanisms of virulence, of this emergent human pathogen.

## Abbreviations

ACT: Artemis Comparative Tool
CARD: Comprehensive Antibiotic Resistance Database
LaESaP: State Laboratory of Public Health of Guanajuato
MCE: Mammalian Cell Entry
NTM: Nontuberculous mycobacteria
RAST: Rapid Annotation using Subsystem Technology
RGI: Resistance Gene Identifier
TDM: Trehalose 6-dimicolate
TMM: Trehalose monomycolate
